# Extra-Limites

**Published:** 1837-07-01

**Authors:** 


### EXTRA-LIMITES.

### MR. ANNESLEY'S REPLY TO THE LATE MR. TWINING.

My attention having recently been called by an Article in the Medico-Chirurgical Review for April 1836, No. 48, page 451, to a work published in Bengal by the late Mr. Twining, in which that gentleman misrepresents my views as to the treatment of diseases of the spleen, I consider it due to the public and to myself to enter into some explanation of that subject.

Mr. Twining's work, I am aware, has been published a considerable time, and I may have been to blame for not having read it, and taken an earlier notice of the remarks, which he has made upon my opinions—my reasons for this were, not that I was unprepared to defend my own position against the attacks of Mr. Twining, for I always intended to do so as soon as I had sufficient leisure for it—but that my important and extensive official duties would have rendered it impossible to follow up the discussion, as I could wish, without trespassing too much upon time, which I could scarcely venture to call my own, looking forward at the same time, to my retirement from the service, at no very distant period, as promising a favourable opportunity for this purpose.

Mr. Twining is now no more, and I would therefore wish to avoid the expression of any feeling his misrepresentation of my views would naturally call forth. Had I been earlier impressed with the nature of these animadversions, I most assuredly should have noticed them before, but, as I have already said, I owe it to the Medico-Chirurgical Review, that I am now acquainted with them, and I cannot, for a moment, submit, to allow the medical public to remain in ignorance of these misrepresentations, or my real views of the treatment of that disease.

Mr. Twining, at page 475—6, declares, that I recommend a free use of mercury as a general practice in diseases of the spleen, and he evidently intends to insinuate, that I advocate the *specific action of mercury* on the constitution in those diseases. This is a mistake on the part of Mr. Twining. I have never advocated any such practice, nor do I intend to do so. He has not, as it appears to me, quoted my words at pages 5 and 6 of my work with fairness, and has drawn an inference from these extracts, which will be found on a reference to the original work, unwarranted. I am always ready to receive with good humour, and indeed with gratitude, the friendly strictures of fair criticism, but without expressing more than unfeigned surprise at such apparently wilful misrepresentation of my views, I here give, in juxta-position, Mr. Twining's garbled extracts and the original paragraphs at pp. 5 and 6 of my work.

1*st Quotation by Mr. Twining.*

"In vol. II. page 5.—In cases of simple tumefaction without inflammatory action, a full dose of calomel at bed-time, and a purgative in the morning, are advisable to be continued daily, or every other day, according to the circumstances of the case."

*The original Paragraph from page* 5, *vol.* II. *Annesley's Diseases of India.*

" When simple tumefaction of the spleen without inflammatory action comes before us, little more is requisite than to carry off the morbid secretions which are usually present, and to give energy to the digestive organs. This is best done by a full dose of calomel at bed-time and the purging powder or bitter purging draught the following morning. These remedies should be continued daily, or every other day, according to circumstances of the case, until the motions (which are morbid in almost all the cases of diseased spleen which have come before us) have assumed a healthy hue. After purgatives have been employed two or three times, then the nitro-muriatic acid wash should be assiduously applied over the region of the spleen, and the nitric acid may be given internally at the same time in the patient's drink."

2*nd Quotation.*

"In congested and tumefied spleen, after having given two or three full doses of calomel, or calomel and opium at bed-time, we should adopt with much benefit, the blue-pill and the aloes and

*Page* 6.

" We cannot too strenuously recommend this treatment in diseases of the spleen, especially the nitro-muriatic solution, but in order that this latter may be beneficial, a continued and well-regulated course of purgatives, and afterwards myrrh pill, giving them every night, and the full dose of calomel every third or fourth night only."

Mr. Twining adds to this—"I am obliged to express my disapprobation of the above instructions in the strongest terms."

of aperients combined with tonics, should be adopted. In cases of congested or tumefied spleen, we should endeavour to support and even to promote the powers of the system, while we purge the bowels; with this view, after having given two or three full doses of calomel, or calomel with opium, at bed-time, we shall adopt with much benefit the blue-pill, and aloes and myrrh pill, giving them every night, and the full dose of calomel every third or fourth night only—but the purgative draught should be continued every morning until the disorder disappears."

Now I ask, is it fair, is it honest, to call this "recommending a free use of mercury, as a general practice, in diseases of the spleen"? Calomel has been recommended by me, exclusively, as a purgative, never as a mercurial to affect the constitution; and there is a wide difference between these two uses of that mineral. I have long observed, and I speak confidently of the fact, that calomel has a specific action upon the tenacious and viscid secretions of the bowels; it attenuates such secretions, and prepares them for being carried off by other purgatives, which would not have been so effectual without the previous dose of calomel; and throughout my work, wherever I have prescribed calomel in full doses, a purgative is always recommended to be taken after a suitable interval. These viscid secretions of the bowels, in which calomel is so useful as a purgative, are particularly common in cases of diseased spleen; hence my recommendation of calomel, as a purgative, in those cases, but, mark, as a purgative only, in aid of those other means suggested for the correction and improvement of the secretions, as stated more fully in my publications—nowhere have I suggested the specific action of mercury upon the constitution in diseases of the spleen, on the contrary, I have warned the practitioner that, under peculiar states of diseased spleen, calomel, even as a purgative, should be used with the greatest caution.—*Vide pp.* 455, 493 *and* 494, *Vol.* II.

From these facts, I am led to believe, that Mr. Twining never read my published opinions, with sufficient care, to enable him to understand what they really were. Had he lived, I would have entered more largely into the subject, but my sole object now, is, to defend my own views from misrepresentation, and to guide the public aright as to the real character of my professional publications. I may have committed errors, none of us are infallible, and I shall feel greatly indebted for any such strictures upon my labours, as will enable me to correct mistakes, or to make my object more intelligible, where the imperfections of my style may have rendered my meaning obscure. Truth is my object, and I am as ready to respect it, under all circumstances, as I am prepared to repel misrepresentation.

(Signed) J. ANNESLEY,

*24th October*, 1836. *Madras Med. Establishment.*

---

### LONDON MISSIONARY SOCIETY.

THE Directors of the London Missionary Society are solicitous to obtain well-qualified medical men, of decided piety, capable of undertaking the most important operations of surgery, to labour as Missionaries in *China*. The situation and circumstances of China, with her vast population and her debasing superstitions, is calculated to excite the sympathies of every Christian mind. The cultivation of an amicable intercourse has been attempted by expensive embassies and commercial energies, but little however has been effected towards the removal of the restrictions on foreigners, or the conciliation of the natives. Missionaries have laboured assiduously, but they have made comparatively little impression. Of late years they have brought medicine to bear, as an auxiliary and as a pioneer, and several benevolent medical men have co-operated with them in their efforts to overcome existing restrictions, and, from those already made, it is very evident that the Chinese esteem most highly our medicines and advice, and place themselves willingly under European practitioners, whose skill and benevolence have been firmly established, and by whose exertions prejudices have been more extensively removed than by all other transactions put together. Dr. A. Pearson introduced vaccination into China in 1805, and the practice soon spread through that vast empire, and is now generally adopted by native surgeons. Drs. Livingstone and Colledge opened an ophthalmic hospital at Macaor in 1827, and, during the last two years, Dr. Parker, an American missionary and physician, has opened a similar institution at Canton, and, during the first nine months, no fewer than 1674 patients were relieved. Dr. P. is still prosecuting his important labours, and is very anxious for others to join him, as spheres are fast opening in which the most talented and zealous may find full scope for their utmost energies. The following medical qualifications would be required:—besides having received a thorough medical education, and possessing extensive practical experience, the candidates should be fully informed on physiology and pathology, therapeutics and pharmacy, clinical and operative surgery, including ophthalmic and aural surgery, with obstetric medicine. Some time will be allowed before leaving England, to be devoted to the acquirement of biblico-theological instruction and the elements of the Chinese language. It is thought that, independent of the immense blessings that may be conferred on China by such medical men, medical science itself must be benefitted by the transmission to this country of much information on a variety of subjects connected with the natural history of plants and of the peculiar diseases of the natives of that interesting portion of the globe.

Further information may be obtained by applying to the Secretaries of the London Missionary Society, at the Mission House, Blomfield Street, Finsbury Circus.

---

### GENERAL REGISTRATION OF DISEASES.

The following address to the members of the profession by the heads of the medical corporate bodies is creditable to the latter, and deserves the attention of the former. We quite agree in all the suggestions which are made.

" We, the undersigned President of the Royal College of Physicians, President of the Royal College of Surgeons, and Master of the Worshipful Society of Apothecaries, having authority from the several bodies whom we represent, do resolve to fulfil the intentions of the Legislature in procuring a better registration of the causes of death, being convinced that such an improved registration cannot fail to lead to a more accurate statistical account of the prevalence of particular diseases from time to time.

We pledge ourselves, therefore, to give in every instance which may fall under our care, an authentic name of the fatal disease.

And we entreat all authorized practitioners throughout the country to follow our example, and adopt the same practice, and so assist in establishing a better registration in future throughout England; for which purpose we invite them to attend to the subjoined explanatory statement, in which they will set forth the provisions of the recent statute, and the means whereby the important object we have recommended may most effectually be attained.

HENRY HALFORD, *President of the Royal College of Physicians.*
ASTLEY COOPER, *President of the Royal College of Surgeons.*
J. HINGESTON, *Master of the Society of Apothecaries.*

*Explanatory Statement.*

The recent Act for registering births, deaths, and marriages in England, presents an opportunity for obtaining that great desideratum in medical statistics—a more exact statement of the causes of death, in the case of every registered death throughout the whole of England and Wales, after the month of June next ensuing.

The register-books in which all deaths are to be registered after the last day of June, 1837, contain columns wherein may be inserted the cause of death, in juxta-position with those other important illustrative circumstances, the sex, the age, and the profession, or calling, of the deceased person. Each register-book will also be assigned to a particular district of small extent, and will thus shew in what part of the kingdom each death has occurred. If, therefore, the cause of death be correctly inserted, there will exist thenceforward public documents, from whence may be derived a more accurate knowledge, not only of the comparative prevalence of various mortal diseases, as regards the whole of England and Wales, but also of the localities in which they respectively prevail, and the sex, age, and condition of life, which each principally affects.

For the attainment of this object it is necessary to insure, as far as it is possible, the 'cause of death.' It is obvious that on this subject the requisite information can seldom be given to the registrar, except by the medical attendant on the deceased person, and that even if the registrar be a medical practitioner (which in many instances will be the case), yet will he often be unable to ascertain the truth in this respect, if he is to depend solely on the reports of persons ignorant of medicine, and of the names and nature of diseases; and it cannot be expected that from his own knowledge he will be able so far to correct their errors as to insure a statement worthy of credit. The requisite information must therefore be supplied, either directly or indirectly, by the medical attendant of the deceased person; that is to say, if such medical attendant is not applied to by the registrar, he must afford the requisite information to those other persons to whom the registrar must apply.

The persons who, according to the act for registering births, deaths, and marriages in England, must give information to the registrar on being requested to do so, are 'some person present at the death, or in attendance during the last illness,' or 'in case of the death, illness, or inability, or default of all such persons, the occupier of the house or tenement, or (if the occupier be the person who shall have died) some inmate of the house or tenement in which such death shall have happened.' It is also provided, that 'for the purposes of this Act, the master or keeper of every gaol, prison, or house of correction, or workhouse, hospital, or lunatic asylum, or public or charitable institution, shall be deemed the occupier thereof.'

It is therefore earnestly recommended that every practising member of any branch of the medical profession who may have been present at the death, or in attendance during the last illness, of any person, shall immediately after such death place in the hands of such other persons as were in attendance, of the occupier of the house in which the death occurred, and of some inmate who may probably be required to give information, written statements of the cause of death, which such persons may show to the registrar, and give as their information on that subject.

It is desirable that such statement should be very short, the column in the register-book in which it is to be inserted being not more than sufficient for the insertion of about ten words of moderate length. It should, therefore, contain only the name of the disease which was considered to be the cause of death, and not a detailed account either of antecedent symptoms, or of the appearances which may have presented themselves after death. It is also desirable that such statement should exhibit the popular or common name of the disease, in preference to such as is known only to medical men, whenever the popular name will denote the cause of death with sufficient precision."

---

### CASE OF EMPYEMA CURED BY PARACENTESIS.

By JAMES EDWARD, M.D., Forfar.

IN May 1836, while Peter Steven, farm servant, aged 22 years, was engaged in unloading a cart, a sack of meal rolled over him and bruised his breast. A short time after this he began to complain of acute pain in the right side, of difficulty in breathing, and a dry cough. His tongue was very foul, his urine small in quantity and highly coloured, his bowels constipated with slight delirium. To relieve him of these, venesection was tried three different times, leeches were applied to the pained part, and tartrate of antimony was given in solution, and blisters and laxatives were used together with antiphlogistic regimen. By these a little temporary relief was afforded. The malady, however, again began and continued, now and then to increase, till December, 1836, when, on comparing the sides of the thorax, I found the right side considerably larger than the left. The intercostal spaces were greatly increased, fluctuation could also be felt, and on applying the ear to the side the respiratory murmur could not be heard, but a dull sound was given by percussion. From these symptoms there was sufficient proof of an accumulation of fluid in the chest. I therefore seriously advised the patient to let me make an opening between the ribs to rid him of this matter. To this proposal he consented with great reluctance. I proceeded in the operation by placing the patient on a chair in the sitting position, and by making an incision, nearly two inches long, upon the upper edge of the eighth rib, rather toward the posterior part of the thorax. I then divided the intercostal muscles, taking care to avoid the intercostal artery which lies in a groove on the lower margin of the seventh rib. By introducing a trocar and canula through the pleura, upwards of three pounds of purulent matter flowed from it at once, and the discharge continued for three months after that time. The wound is now quite whole, and the side appears to be smaller than the other. By percussion the sound is dull, and the respiratory murmur cannot be heard. He has now quite overcome the disease, and is enjoying a tolerable degree of health. The only remedies used after the operation was a nourishing diet and mineral acids.

PRINCIPAL ORIGINAL PAPERS PUBLISHED IN THE WEEKLY AND MONTHLY BRITISH JOURNALS, BETWEEN THE 25TH OF MARCH AND THE 10th JUNE, 1837.

1. ANATOMY.

*Dublin Journal.*

On the Discovery of Ciliary Motions in the Cavities of the Brain; by Purkinge.—March.

On the Organized Bodies found in the Seminal Fluid of Animals, and their Analogy to the Pollen of Plants; by G. R. Treveranus.—March.

2. MATERIA MEDICA, AND PRACTICE OF MEDICINE.

*Dublin Journal.*

Medical Problems. By William Griffin, M.D. Limerick.—March.

Scarlatina, with Ulceration of the Throat, and inflammation of the arachnoid membrane. By T. Burke, Esq.—March.

History of an extraordinary case which terminated fatally in the Hospital of the 2d Dragoon Guards, at Cahir Barracks, 30th April, 1829. By Andrew Brown.—May.

On the use of the nitro-muriatic acid Bath. By Charles Lendrick, M.D.—May.

On the treatment of various diseases by means of creosote. By Sir Francis Smith, M.D.—May.

*Medical Gazette.*

On the curative effects of the strychnos nux vomica in dyspepsia. By H. S. Belcombe, M.D.—March, 25.

Remarks on the treatment of hydrocephalus acutus. By Dr. Mayo.—April 8.

On the late influenza. By Dr. Heberden. —April 8.

On the application of solid nitras argenti in the gonorrhœa of women. By A. Hannay, M.D.—May 6.

Case of diabetes; with remarks. By Henry Snowden, Esq.—April 22.

Case of severe cough ending in rupture of the lung, and general emphysema of the body. By T. G. Hicks, Esq.—April 22.

On the bruit du diable. By E. Boult, Esq. —April 22.

Case of cephalœa from a tubercle embedded in the cerebellum. By R. Jeffreys, Esq.— May 20.

Case of laryngitis and tracheotomy. By T. Armstrong, Esq.—May 20.

Case in which seven half-crowns had been swallowed. By H. Wakefield, Esq.— May 20.

On the inapplicability of statistics to the practice of medicine. By M. Double.— June 3.

Case of spontaneous mortification of the toe, treated by tightly bandaging the leg. By T. Spender, Esq.—May 13.

On lytta in diabetes. By Jesse Leach, Esq. May 13.

Observations on the stethoscope. By Geo. Budd, Esq. May 27.

On the tonic treatment of erysipelas. By A. Kirhup, Esq.—April 29.

Clinical instruction.—April 29.

On the late influenza. By E. Greenhow, M.D.—April 1.

Cold sponging in fits. By C. J. Aldis, Esq. April 1.

On paralytic and painful nervous affections. By T. Grantham, Esq.—April 15.

On the epidemic influenza. By G. Fife, M.D.—April 15.

On the treatment of inflammation, with some remarks on influenza. By H. Searle, Esq.—April 15.

*Lancet.*

Case of poisoning with aconite, successfully treated. By H. C. Sherwin, Esq., Surgeon, Hull. March 25.

Account of an epidemic erysipelas at West Auckland and its neighbourhood. By John Kilburn, Esq.—May 20.

3. PHYSIOLOGY.

*Medical Gazette.*

An experimental enquiry into the comparative state of urea in healthy and diseased urine, &c. By R. M'Gregor, Esq.— May 20.

On the organic filament and its tissues. By T. Hake, M.D.—April 1.

On the influence of gravity on the circulation of the blood. By F. Mosely, Esq. —April 15.

What are the practical indications of the pulse in disease? By R. Burke, M.D. Ap. 8.

On the connection of the anterior columns of the spinal chord with the cerebellum. By Samuel Solly, Esq.—April 8.

On the powers on which the functions of life in the more perfect animals depend, and on the manner with which they are associated in the production of their more complicated results. By R. Philip, M.D. —March 25.

On the mechanism of respiration, and the respiration of the fœtus. By M. W. Hilles, Esq.—May 20.

4. SURGERY.

*Dublin Journal.*

Remarks on fracture of the neck of the thigh bone, external to the capsule. By James Douglas, M.D. March.

An essay on wounds of the heart. By Cathcart Lees, A B —May.

*Medical Gazette.*

Hernia—rare form of stricture. By J. Adams, Esq.—April 22.

Account of a new speculum vaginæ. By W. Beaumont, Esq.—April 22.

Two lectures on lithotrity, and the bilateral operation. By Edwin Lee, Esq.—May 13.

On the organization and economy of bone. By Prof. Stanley.—June 10.

Solid nitrate of silver in gonorrhœa. By James Smith, M.D.—May 27.

On phlegmasia dolens of the eye. By R. Middlemore, Esq.—April 1.

Practical Observations on Hernia. By C.F. Skey, Esq.—April 1.

*Lancet.*

Dislocation of the radius on the posterior surface of the ext. condyle of the humerus. By G. Hopper, Esq. Surgeon.—April 15.

On the operation for strangulated hernia, to facilitate the taxis, without wounding the peritoneum. By M. W. Hilles, Esq. Surgeon.—April 15.

On the cure of club-foot by division of the tendo-achillis. By Dr. W. J. Little.—April 15.

5. PATHOLOGY.

*Dublin Journal.*

On the occurrence of crystals in the intestinal canal, in cases of abdominal typhus. By Professor Schoenlean, of Zurich.—March.

An account of tubercles in the air-cells of a bird, and some observations on tubercles in general. By Robert Harrison, M.D. —May.

*Medical Gazette.*

On the crepitous râles. Strictures on certain passages of M. Laennec's work. By E. Harrison, M.D. May 6.

On the acoustic principles of the stethoscope. By Charles Williams, M. D.—June 3.

On the connexion of urea with gout. By G. Weatherhead, Esq. M.D.—May 27.

On the bruit du diable. By T. Ward, M.D. April 1.

Hydatid tumors—their formation and pathology. By H. C. Sherwin, Esq.—Apr. 1.

*Lancet.*

On the connexion between hectic fever and suppuration, and the absorption of pus. By C. W. Edwards, Esq.—April 22.

6. MIDWIFERY.

*Dublin Journal.*

On the length of the umbilical cord, and its influence upon parturition. By F. Churchill, M.D.—March.

Observations on the artificial dilatation of the mouth of the womb during labour, and upon instrumental delivery, &c. By Robert Collins, M.D. March.

An enquiry into the management of the first stage of labour. By Edw. Murphy, A.M. M.D.—May.

*Medical Gazette.*

Advantage of ergot of rye over instrumental assistance in some cases of labour. By J. Evans, Esq.—April 22.

On the first stage of labour. By James Hamilton, M.D. June 10.

Remarks on a case published in the Medical Gazette, entitled "Pregnancy, with imperforate Uterus" By J. North, Esq. —June 10.

*Lancet.*

On the prevention of anticipated uterine hæmorrhage after parturition. By Tho. Abraham, Esq.—April 22.

Remarks on the employment of the compress and bandage for the suppression of uterine hæmorrhage after delivery. By W. A. Walford, Esq.—April 22.

6. LECTURES.

Lectures on materia medica, or pharmacology and general therapeutics, delivered at the Aldersgate School of Medicine. By John Pereira, F.R.S.—*Med. Gazette*

Clinical lectures delivered at the Meath Hospital and County of Dublin Infirmary, during 1836-7. By Prof. Graves. —*Medical Gazette.*

Abstract of lectures delivered before the College of Surgeons, in April, 1837. By Professor Stanley.—*Medical Gazette.*

Course of lectures on medical jurisprudence. By Professor A. T. Thomson.—Now being delivered at the University of London.—*Lancet.*

Course of lectures on the physiology of the nervous system. By M. Magendie. Delivered at the College of France in 1836. —*Lancet.*

Course of lectures on materia medica and therapeutics. By Dr. Symond.—*Lancet.*

Clinical lecture on the exanthematic form of syphilis. By Dr. Wallace.—*Lancet.*

Notes of lectures on physiology. Delivered at the Argyle Rooms. By Dr. Fletcher. —*Medical and Surgical Journal.*

